# The quality of bone and paraspinal muscle in the fragility osteoporotic vertebral compression fracture: a comprehensive comparison between different indicators

**DOI:** 10.1186/s12891-024-07587-8

**Published:** 2024-06-15

**Authors:** Sizheng Zhan, Haoning Ma, Xingguang Duan, Ping Yi

**Affiliations:** 1 Department of Spine Surgery, China, Japan Friendship Hospital, No.2 Yinghuayuan East Street, Chaoyang District, Beijing, 100029 China; 2 School of Mechatronical Engineering, Beijing Institute of Technology, Beijing, 100081 China

**Keywords:** Osteoporotic vertebral compression fracture, Vertebral bone quality score, Paraspinal muscle, CT-based Hounsfield unit value

## Abstract

**Purpose:**

To evaluate the value of five indicators in predicting OVCF through a retrospective case–control study, and explore the internal correlation of different indicators.

**Method:**

We retrospectively enrolled patients over 50 years of age who had been subjected to surgery for fragility OVCF at China Japan Friendship Hospital from January 2021 to September 2023. Demographic characteristics, T-score based on dual-energy X-ray absorptiometry (DXA), CT-based Hounsfield unit (HU) value, vertebral bone quality (VBQ) score based on magnetic resonance imaging (MRI), relative cross-sectional area (rCSA) and the rate of fat infiltration (FI) of paraspinal muscle were collected. A 1:1 age- and sex-matched, fracture-free control group was established from patients admitted to our hospital for lumbar spinal stenosis or lumbar disk herniation.

**Results:**

A total of 78 patients with lumbar fragility OVCF were included. All the five indicators were significantly correlated with the occurrence of OVCFs. Logistic regression analysis showed that average HU value and VBQ score were significantly correlated with OVCF. The area under the curve (AUC) of VBQ score was the largest (0.89). There was a significantly positive correlation between average T-score, average HU value and average total rCSA. VBQ score was significantly positive correlated with FI.

**Conclusion:**

VBQ score and HU value has good value in predicting of fragility OVCF. In addition to bone mineral density, we should pay more attention to bone quality, including the fatty signal intensity in bone and the FI in paraspinal muscle.

## Introduction

Fragility fractures refer to fractures that occur after no trauma or minor trauma [1]. The most common fragility fracture is the osteoporotic vertebral compression fracture (OVCF) [2]. With the aging of the population, the incidence of fragile OVCF is increasing. According to the results of the osteoporosis epidemiological survey in China from 2017 to 2018 [3], the prevalence of OVCF in the population was 10.5% in men and 9.7% in women over 40 years old. The OVCF is one of the main causes of disability and death in elderly patients, and also causes a heavy burden to the family and society [4]. Therefore, it is of great significance to screen and identify high-risk factors for fragile OVCF in advance and provide timely preventive measures.

The most common cause of fragility OVCF is osteoporosis. Currently, dual-energy X-ray absorptiometry (DXA) is the gold standard for assessing bone mineral density (BMD), but its measurements are often overestimated due to lumbar degeneration, such as the formation of vertebral osteophytes [5]. Schuit [6] found that more than half of all fragility fractures occur in patients diagnosed without osteoporosis by DXA. Therefore, BMD measured by DXA may not be a sensitive indicator for fragility OVCF.

In order to enhance the assessment of vertebral bone quality in patients, numerous indicators have been proposed by scholars, including CT-based Hounsfield unit (HU) value [7, 8] and vertebral bone quality score (VBQ) based on magnetic resonance imaging (MRI) [9, 10]. Compared with DXA, HU value and VBQ score are easy to obtain in clinic, and do not need to add additional examination for patients. The research results of Zou et al. showed that low HU value was related to the occurrence of lumbar OVCF [7]. The research of Li et al. showed that VBQ can be used to predict the occurrence of OVCF [10]. Meanwhile, with the recognition of the importance of paraspinal muscle in maintaining lumbar spine stability and reducing lumbar spine load [11, 12], researchers [13, 14] found that the cross-sectional area (CSA) of paraspinal muscle in OVCF patients was smaller and the rate of fat infiltration (FI) was higher.

Therefore, we intend to evaluate the value of different indicators in predicting OVCF through a retrospective case–control study, and explore the internal correlation of different indicators.

## Method

### Patients

Prior to the start of the study, the protocol was approved by the ethics review committee of China Japan Friendship Hospital. We reviewed patients admitted to our department of spinal surgery from January 2021 to September 2023. Inclusion criteria were (1) age $\ge$ 50 years, regardless of the gender; (2) completed perioperative DXA scans, lumbar MRI and lumbar CT scans within 1 weeks; (3) fresh lumbar fragility OVCF; (4) the time between back pain onset and surgery is less than 2 weeks. Exclusion criteria were (1) previous history of fracture, surgery, trauma, or infection in L1 − L4; (2) Kummell’s disease; (3) long-term oral hormone-like drug therapy; (4) high-energy vertebral fractures, such as car accidents, falls, etc. In addition, for comparison with patients who did not have fractures, a 1:1 age- and sex-matched, fracture-free control group was established from patients admitted to our hospital for lumbar spinal stenosis or lumbar disk herniation with or without degenerative lumbar spondylolisthesis (the degree is less than 50%). The exclusion criteria were as described above.

### Variables

We collected demographic characteristics of patients via our hospital’s electronic medical record system, including age, sex, body mass index (BMI), course of disease (the time between back pain onset and surgery), and comorbidities, etc.

### BMD evaluation

For BMD, DXA scans (Discovery A densitometers, Hologic Inc., Bedford, MA, USA) of lumbar, femur and hip were performed during hospitalization. The T-score of L1-L4 were recorded from DXA. T-score of the fractured vertebrae were discarded.

$$average\ T-score=^{T-score\ of\text{ unfractured vertebrae}(\text{L}1-\text{L}4) }\!\left/ \!_{3}\right.$$

### Calculation of the VBQ score

**Fig. 1:**
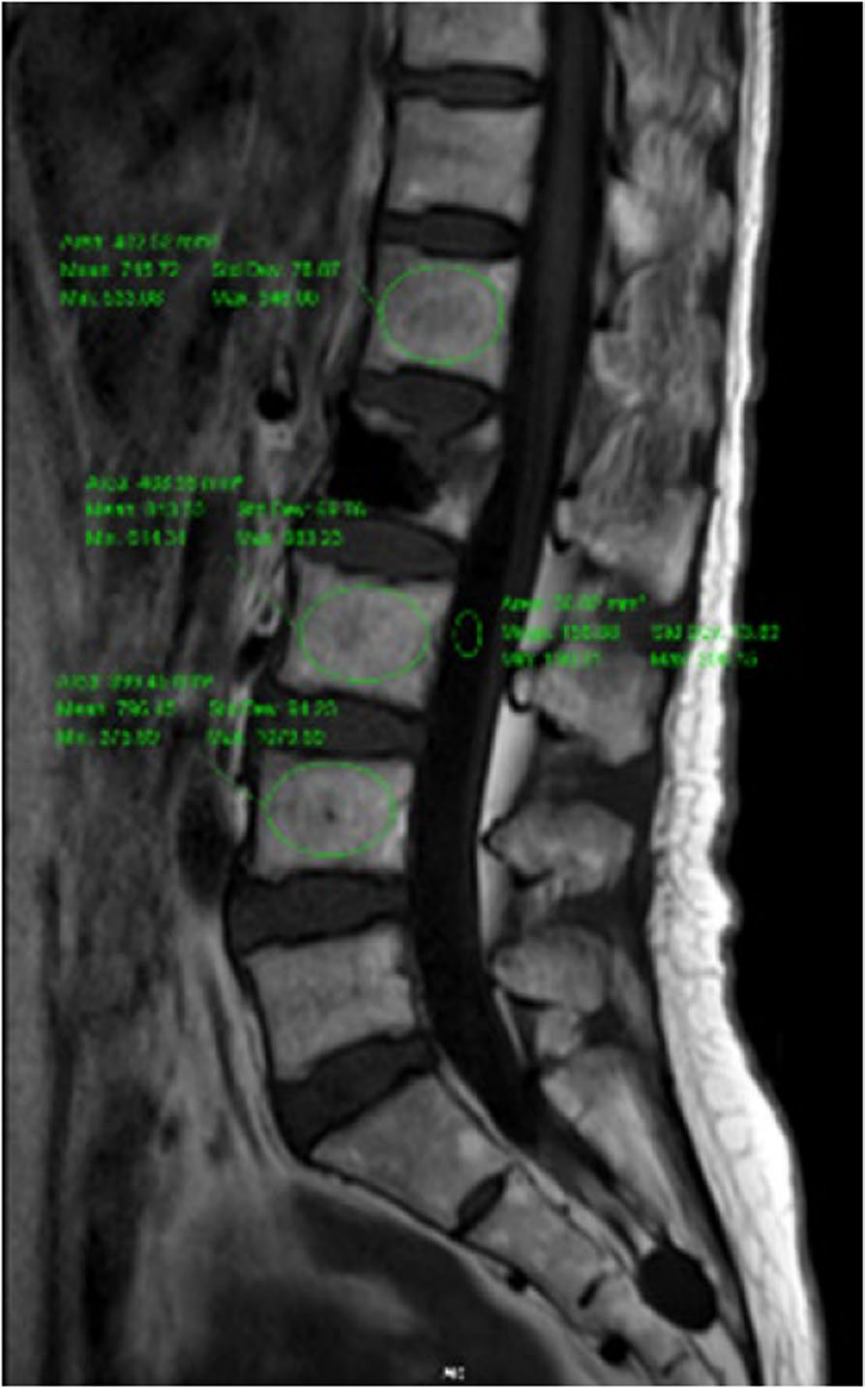
Non-contrast-enhanced T1-weighted MRI image shows the SI of L1, L3, L4 and SI of CSF using ROIs

According to the method proposed previousl [15], the VBQ score was calculated using signal intensity (SI) obtained from a non-contrast, T1-weighted lumbar spine MRI image (Fig. 1). Firstly, the mean SI of the trabecular bone of the vertebrae (L1 − L4) was measured using the midsagittal slices. For patients with abnormalities in the midsagittal slice (eg, hemangioma, venous plexus, scoliosis changes), measurements were performed using the parasagittal slices. The fractured vertebrae was excluded from the calculation and only the remaining vertebrae were used to calculate the VBQ score. The median SI of the L1-L4 was then divided by the SI of the cerebrospinal fluid (CSF) at L3 level to obtain the VBQ score. CSF ROI was placed at L2 or L4 level when L3 CSF space was completely blocked by descending nerve roots.

$$VBQ=^{Median(SIL1-L4)}\!\left/ \!_{SIL3CSF}\right.$$

### The HU values of vertebrae in CT

**Fig. 2:**
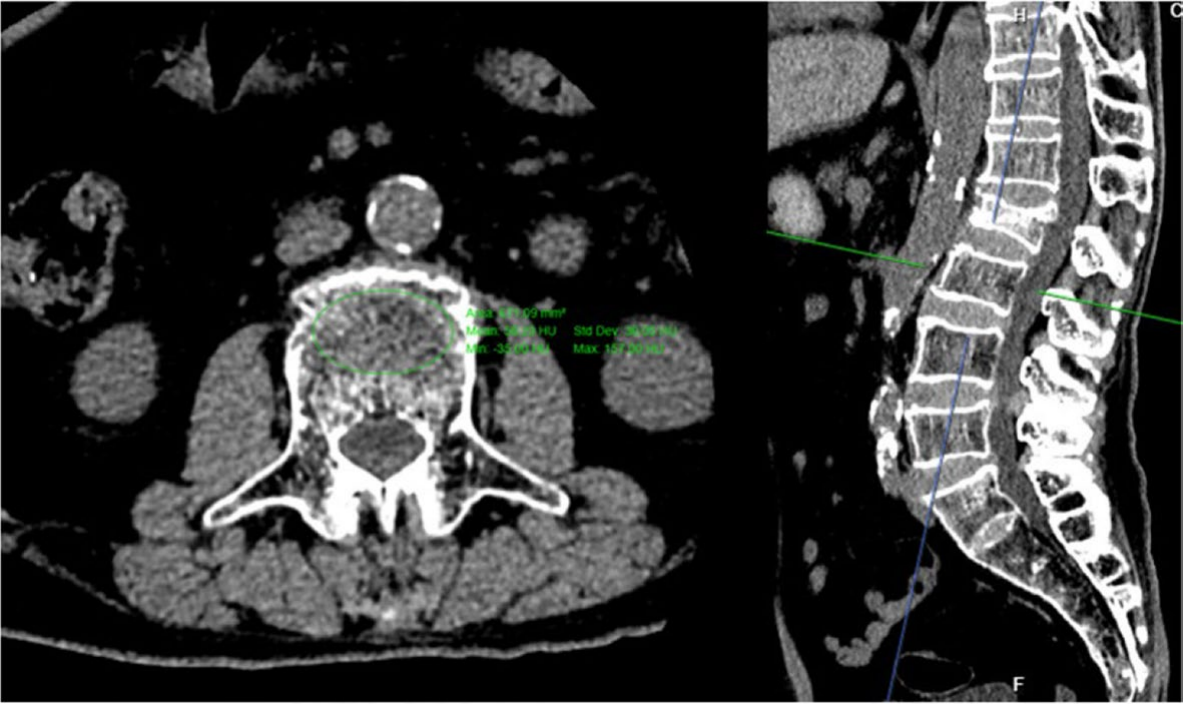
Example of the measurement of HU value at L3

The HU values of L1-L4 were measured for each patient according to the method of previous studies (Fig. 2) [8]. An oval region of interest inclusive of trabecular bone was placed in the middle-axial CT image of vertebral body. We avoided placing the ROI near areas that would distort the BMD measurement (posterior venous plexus; focal heterogeneity or lesion, including compression fracture; and imaging-related artifacts).

$$average\ HU=^{HU \,of\text{ unfractured vertebrae}(\text{L}1-\text{L}4) }\!\left/ \!_{3}\right.$$

### Paraspinal muscle evaluation on MRI

**Fig. 3:**
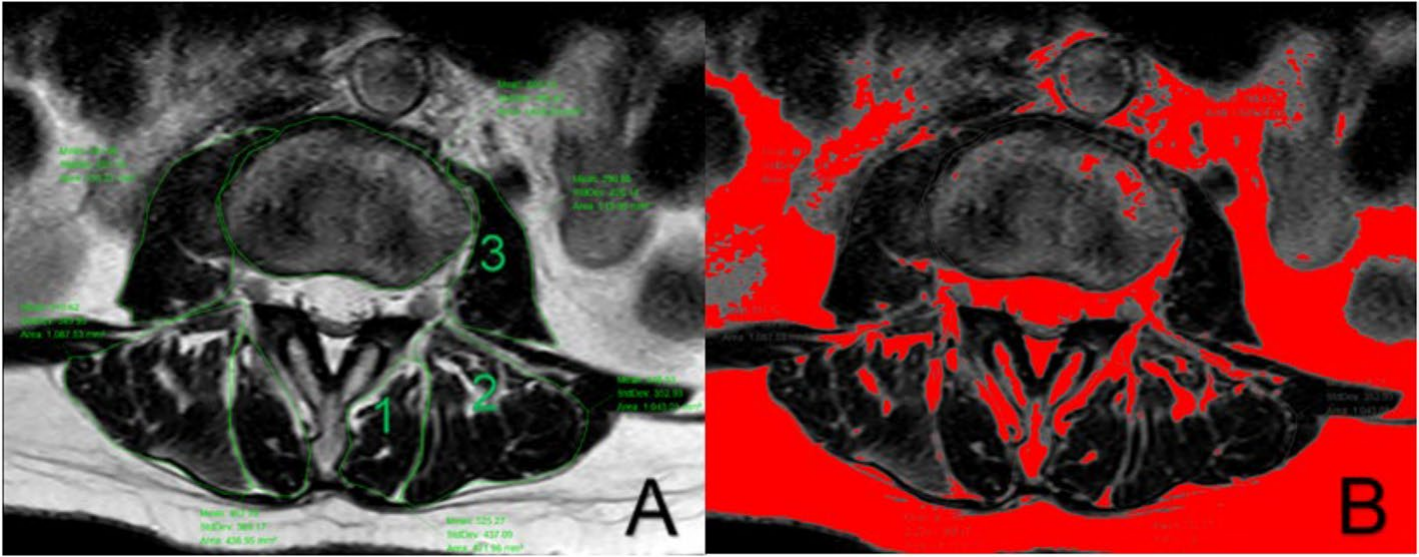
**A** Measurements of paraspinal muscular parameters on axial T2-weighted MRI. Regions of total cross-sectional area of multifidus (1), erector spinae (2), psoas muscle (3) level were outlined by green lines; **B** Thresholding technique to highlight intramuscular fat area (red area)

The Picture Archiving and Communication Systems (PACS) processed imaging data of the paravertebral muscles and measured the cross-sectional area (CSA) of the paraspinal muscles. Relative cross-sectional area (rCSA, the ratio of cross-sectional area of muscle to that of disc at the same level) was introduced for reducing the effect of body shape on muscular parameters. The fat infiltration (FI) of the paraspinal muscles (psoas major [PM], multifidus [MF] and erector spinae [ES]) were measured by Image J software (Fig. 3). Middle images of each disc space at L1-2, L2 − 3, L3 − 4, and L4 − 5 was selected by locating lines in the sagittal slice. The pseudocoloring technique was used to measure the percentage of fat content. The bright pixels of fatty tissue are painted red and the percentage of red areas in the muscle compartment was calculated [16]. The paraspinal muscle-related parameters were the FI averaged on both sides and the three slices and the average total rCSA of PM, MF and ES across different cross Sects [17].

All quantitative measurements were analyzed using the average values of the measurements obtained by the two investigators.

### Statistical analysis

Statistical analysis was performed using IBM SPSS Statistics for Windows, version 26.0 (IBMCorp., Armonk, N.Y., USA). The age- and sex-matching process was performed with the case–control matching function of SPSS. Inter-rater and intra-rater reliability was assessed with the intraclass correlation coefficient (ICC), respectively. Paired t test was used to compare the difference between the fracture group and the control group without fractures. The correlation analysis was calculated using Spearman correlation coefficients. Logistic regression analysis was used to examine the contribution of T-score, paravertebral muscle-related parameters, VBQ score and Hu values to the OVCF. Receiver-operator curve (ROC) was used to evaluate the performance of T-score, paravertebral muscle-related parameters, VBQ score and Hu values in distinguishing fracture patients from control patients. Statistical significance was set at *P* value < 0.05. STATA 15.0 were used to draw the ROC curves.

## Results

**Fig. 4:**
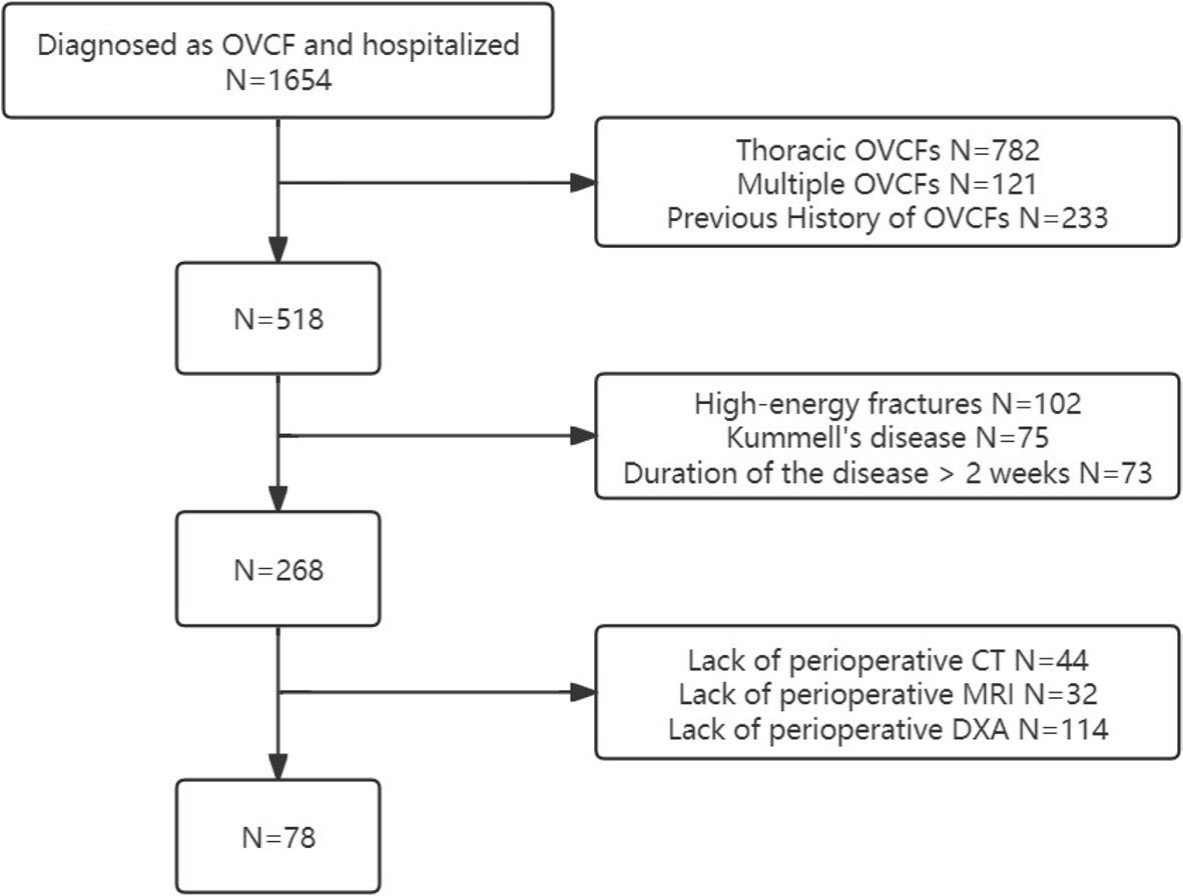
Patient flow

A total of 78 patients with lumbar fragility OVCF were included in this study, including 28 males and 50 females (Fig. 4). As for the 1:1 age and sex matched patients, all patients with OVCF were successfully matched to patients without fractures, and 78 control patients were also included in the study. There were no significant differences in gender and age between the two groups (Table 1). The ICCs of HU value, VBQ score and paraspinal muscle related parameters were all above 0.8.

**Table 1:** Patient demographics and OVCF indicators

	**OVCF (*n* = 78)**	**non-OVCF (*n* = 78)**	***P* value**
**Age**	72.21 + 10.53	71.57 + 9.42	0.666
**Gender**	50 (64.1%)	50 (64.1%)	1
**BMI**	22.79 + 3.08	22.91 + 2.86	0.806
**Hypertension**	42	35	0.262
**Diabetes**	46	37	0.149
**Hyperlipidemia**	31	38	0.259
**L1 fracture**	23 (29.5%)	-	-
**L2 fracture**	18 (23.1%)	-	-
**L3 fracture**	26 (33.3%)	-	-
**L4 fracture**	11 (14.1%)	-	-
**Average T**	(-2.47 + 0.83)	(-1.34 + 1.44)	< 0.001
**Average HU**	66.96 + 10.3	88.74 + 16.32	< 0.001
**VBQ**	4.13 + 0.34	3.06 + 0.39	< 0.001
**Average total rCSA**	2.48 + 0.29	2.69 + 0.44	0.001
**Average FI**	16.18 + 1.14	17.18 + 0.95	< 0.001

**Fig. 5:**
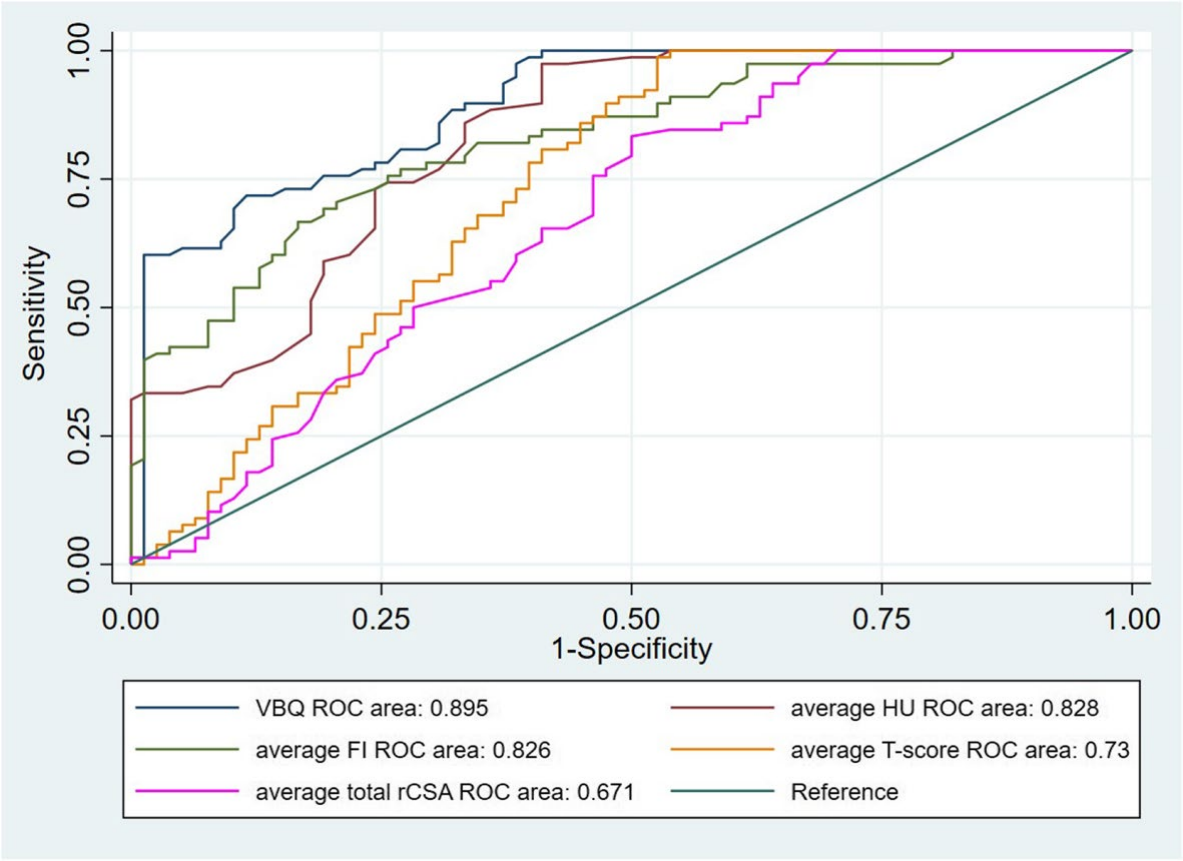
The ROC curves of the five indicators for OVCF

The average T-score, VBQ score and average FI in the OVCF group were significantly higher than those in the control group, and HU value and average total rCSA were significantly lower than those in the control group. T-score, average HU value, VBQ score, average total rCSA and average FI were all significantly correlated with the occurrence of OVCFs. The correlations were ranked from high to low as VBQ score, average HU value, average FI, T-score, and average total rCSA (Rho = 0.84, -0.665, 0.568, -0.399, -0.297, respectively; Table 2). After adjusting for age, sex and BMI, logistic regression analysis showed that HU value and VBQ score were significantly correlated with OVCF (OR = 0.39, 95%CI: 0.29–0.77; and OR = 2.98, 95%CI: 1.78–8.25, respectively; Table 2). ROC curve was used to evaluate the different indicators for predicting OVCF. The area under the curve (AUC) of VBQ score was the largest (0.895, Fig. 5).

**Table 2:** Sperman correlation analysis and logistics regression used to evaluate the correlation between the five indicators and OVCF

	**Rho**	***P***	**OR**	**95% CI**	***P***
**T-score**	-0.399	< 0.001	0.533	0.37–1.13	0.08
**HU**	-0.665	< 0.001	0.39	0.29–0.77	0.02
**VBQ**	0.84	< 0.001	2.98	1.78–8.25	0.007
**CSA**	-0.297	0.007	0.69	0.52–1.05	0.91
**FI**	0.568	< 0.001	1.47	0.85–2.3	0.18

The correlation analysis among the different indicators found that there were significant correlations among the five indicators (Table 3). There was a positive correlation between average T-score, HU value and average total rCSA. VBQ score was significantly positive correlated with FI. The strongest correlations were HU value and average T-score (Rho = 0.926), VBQ score and average FI (Rho = 0.919), respectively.

**Table 3:** Sperman correlation analysis used to evaluate the correlation between the five indicators

	**T-score**	**HU**	**VBQ**	**rCSA**	**FI**
**T-score**		0.926	-0.82	0.679	-0.759
**HU**	0.926		-0.823	0.625	-818
**VBQ**	-0.82	-0.823		-0.634	0.919
**rCSA**	0.679	0.625	-0.634		-0.74
**FI**	-0.759	-818	0.919	-0.74	

## Discussion

In the face of an increasing number of risk indicators for OVCF, we compared five indicators that have been widely studied firstly. Through the retrospective case–control study, we found that all five indicators were associated with OVCF, and the two most strongly correlated indicators were VBQ score and HU value. At the same time, the internal correlation analysis showed that T-score, HU value and rCSA were significantly positive correlated, and FI and VBQ score were significantly positive correlated.

It is of great significance to screen and identify high-risk factors for fragile OVCF in advance and provide timely preventive measures. The most common cause of fragility OVCF is osteoporosis. Currently, dual-energy X-ray absorptiometry (DXA) is the gold standard for assessing bone mineral density (BMD), but its measurements are often overestimated due to lumbar degeneration, such as the formation of vertebral osteophytes [5]. With increasing awareness of identifying high risk factors for OVCF, it has been identified based on some routine tests. In 2013, it was found that CT-based HU value could be used to determine BMD [8]. A number of studies [7, 18, 19] by Li have shown that HU value has good application value in identing the high-risk factors of OVCF and postoperative nonunion. A wide range of studies have confirmed that HU value can be used as a simple and important method to evaluate BMD in patients [19].

VBQ score based on MRI is a new technique to evaluate bone quality first introduced by Ehresman in 2019 [20], and its principle is to measure the fat content of the vertebra and indirectly reflect the bone quality [20]. A good correlation between the VBQ score and BMD has been widely demonstrated [21]. It has also been found to predict complications after spinal surgery [22]. A retrospective single-center cohort study also found that the VBQ score could predict fragile vertebral fractures independently of BMD [23].

With the deepening of clinical research, clinicians gradually discovered the important role of paraspinal muscle. A large number of studies have shown that the reduction of paraspinal muscle CSA and the increase of FI are closely related to the occurrence of various lumbar diseases [14, 17, 24]. Paraspinal muscle-related parameters were significantly correlated with BMD [11]. Paraspinal muscle degeneration can be used to predict OVCF [14], postoperative outcome of lumbar stenosis [24], postoperative complications of scoliosis [17], etc. The clinical outcome of patients can be improved by strengthening the paraspinal muscle [25].

Each indicator has been validated in clinical studies for its important role in assessing risk factors for OVCF. But which indicator is better, the internal correlation between each indicator is still questionable. In our study cohort, VBQ score and HU value ranked first and second in evaluating OVCF risk, superior to T-score. Therefore, in clinical work, we recommend the priority selection of VBQ score as an evaluation indicator for OVCF risk. If the patient cannot complete MRI examination, we suggest that HU value should be selected as an evaluation indicator to identify the risk of OVCF. Compared with DXA, CT or MRI can also provide clinical information in addition to bone quality. At the same time, Ehresman’s study [23] showed that the fatty signal intensity in bone has an important influence on bone quality. In our study, we also found that in addition to BMD, the fatty signal intensity in bone may be more important.

In the evaluation of paraspinal muscle, we found that FI was more important than CSA. Other studies [14] have come to similar conclusions that OVCF is more related to FI than CSA. We have chosen rCSA to reduce the interference caused by factors such as height, but the conclusion is still consistent. The possible reason is that greater CSA does not mean stronger paraspinal muscle strength, and the correlation between paraspinal muscle strength and FI is stronger. At the same time, we found that the correlation between OVCF and FI or CSA was not as good as that of VBQ score, HU value or T-score. This suggests that OVCF may be more related to bone quality rather than paraspinal muscles. Finite element analysis found that strengthening the paravertebral muscle could only reduce the vertebral load by 3.1% at most^12^. Therefore, as for the prevention of OVCF, the most noteworthy is the bone quality. The paraspinal muscle is also a very critical intervention variable.

As for the internal correlation of different indicators, we found that T-score was significantly correlated with HU value, and VBQ score was significantly correlated with FI. The results from other studies [7, 11] also show that T-score and HU are significantly consistent. The measurement of the two depends on bone trabecular density, etc. So in the clinic, HU value may replace T-score to assess the BMD. Li’s study^16^ showed that the CSA and FI of the paraspinal muscle may be more likely to influence the value of the VBQ score than BMD, which showed the important influence of the fatty signal intensity in bone quality.

The limitations of this study should also be considered. First, as a retrospective study, the data were obtained from the medical record system. Some data, such as patients’ special comorbidities, smoking or drinking history, were not fully recorded. Some studies have proved that these comorbidities might affect the occurrence of OVCF. Second, patients in the control group were hospitalized for surgical treatment due to lumbar spinal stenosis or lumbar disc herniation. Lumbar MRI scans are less common in patients treated conservatively. Therefore, VBQ scores and the other indicators in patients who did not receive any surgical treatment were not analyzed in this study. In addition, the acquisition of HU value, VBQ score and paraspinal muscle-related parameters differ greatly. Although there is good internal consistency in our study, this is based on sufficient sample size training and internal communication among data collectors.

## Conclusion

VBQ score and HU value has good value in predicting of fragility OVCF. The higher the VBQ score, the lower the HU value, the greater the likelihood of a fragility OVCF. In addition to BMD, we should pay more attention to bone quality, including the fatty signal intensity in bone and the FI in paraspinal muscle.

## Data Availability

Almost all data generated or analysed during this study are included in this published article. The datasets used and/or analysed during the current study available from the corresponding author on reasonable request.

## Abbreviations

OVCF: Osteoporotic vertebral compression fracture
DXA: Dual-energy X-ray absorptiometry
HU: Hounsfield unit
VBQ: Vertebral bone quality
MRI: Magnetic resonance imaging
rCSA: Relative cross-sectional area
FI: Fat infiltration
AUC: Area under the curve
BMI: Body mass index
SI: Signal intensity
CSF: Cerebrospinal fluid
BMD: Bone mineral density
